# Efficient small fragment sequencing of human, cattle, and bison miRNA, small RNA, or csRNA-seq libraries using AVITI

**DOI:** 10.1186/s12864-024-11013-7

**Published:** 2024-11-29

**Authors:** Anna L. McDonald, Andrew M. Boddicker, Marina I. Savenkova, Ian M. Brabb, Xiaodong Qi, Daniela D. Moré, Cristina W. Cunha, Junhua Zhao, Sascha H. Duttke

**Affiliations:** 1 School of Molecular Biosciences, College of Veterinary Medicine, Washington State University, Pullman, WA USA; 2 Element Biosciences, San Diego, CA USA; 3 Animal Disease Research Unit, Agricultural Research Service, United States Department of Agriculture, Pullman, WA 99164 USA; 4 Department of Veterinary Microbiology and Pathology, Washington State University, Pullman, WA 99164 USA

**Keywords:** Small RNA sequencing (sRNA-seq), Capped small RNA sequencing (csRNA-seq), AVITI, Illumina, Livestock

## Abstract

**Background:**

Next-Generation Sequencing (NGS) catalyzed breakthroughs across various scientific domains. Illumina’s sequencing by synthesis method has long been central to NGS, but new sequencing methods like Element Biosciences’ AVITI technology are emerging. AVITI is reported to offer improved signal-to-noise ratios and cost reductions. However, its reliance on rolling circle amplification, which can be affected by polymer size, raises questions about its effectiveness in sequencing small RNAs (sRNAs) such as microRNAs (miRNAs), small nucleolar RNAs (snoRNAs), and many others. These sRNAs are crucial regulators of gene expression and involved in various biological processes. Additionally, capturing capped small RNAs (csRNA-seq) is a powerful method for mapping active or “nascent” RNA polymerase II transcription initiation in tissues and clinical samples.

**Results:**

Here, we report a new protocol for seamlessly sequencing short fragments on the AVITI and demonstrate that AVITI and Illumina sequencing technologies equivalently capture human, cattle (*Bos taurus*), and bison (*Bison bison*) sRNA or csRNA sequencing libraries, increasing confidence in both sequencing approaches. Additionally, analysis of generated nascent transcription start site (TSS) data for cattle and bison revealed inaccuracies in their current genome annotations, underscoring the potential and necessity to translate small and nascent RNA sequencing methodologies to livestock.

**Conclusions:**

Our accelerated and optimized protocol bridges the advantages of AVITI sequencing with critical methods that rely on sequencing short fragments. This advance bolsters the utility of AVITI technology alongside traditional Illumina platforms, offering new opportunities for NGS applications.

**Supplementary Information:**

The online version contains supplementary material available at 10.1186/s12864-024-11013-7.

## Background

Next-generation sequencing (NGS) revolutionized biology and biomedicine and has led to considerable advancements in research, clinical diagnostics, and agricultural and environmental applications. Recent key contributing factors included cost-efficient sequencing, greater accessibility for researchers and clinicians, increased speed, throughput, and precision. Simultaneous analysis of numerous sequences facilitated the identification of genetic variants, aiding the understanding of diseases, population genetics, breeding, and evolutionary studies.

The Illumina sequencing by synthesis method has long been a cornerstone in NGS, but new technologies are emerging. Recently, Element Biosciences released the AVITI platform. Instead of linear library amplification and sequencing by fluorescently-labeled and reversibly-terminated nucleotides, as done by Illumina instruments, AVITI circularizes library molecules and uses rolling circle amplification, followed by sequencing using specific detector molecules called avidites. As these multivalent molecules are highly specific and bind multiple extension sites within an amplified “polony”, AVITI requires lower reagent concentrations, which translates into low sequencing costs and less background signal [1]. However, DNA circularization may be size-dependent and generally inefficient for shorter polymers, depending on circularization mechanism, especially below 150 bp [2–4].

Small RNAs (sRNAs) such as microRNAs (miRNAs), small nucleolar RNAs (snoRNAs), small interfering RNAs (siRNAs), piwi-interacting RNAs (piRNAs), and many others [5–8], are crucial regulators of gene expression and are involved in various biological processes, including development, defense against viruses and transposons, and maintenance of genome stability [4, 9–11]. Consequently, they are a fundamental area of study in molecular biology and a focus in the search for future therapeutic interventions, diagnostics, and crop improvements. In addition, capturing capped small RNAs (csRNA-seq) has emerged as a powerful method to identify sites of active or “nascent” transcription from total RNA or clinical samples [12–14]. Circulating cell-free DNA (cfDNA), which is typically around 180–200 bp in length, are also of emerging interest for diagnostics, disease monitoring, and therapeutic applications [15]. We therefore compared the AVITI and Illumina sequencing technologies in their ability to sequence small fragment libraries.

Here we present an expedited and refined protocol for short fragment sequencing with AVITI, which aligns seamlessly with commercially available sRNA library kits. We show that sequencing short fragments like sRNAs (18–60 nt in size) or initiating RNA polymerase II transcripts (csRNA-seq) [13] gives uniform results with AVITI and Illumina sequencing technologies. Moreover, generation of sRNA and the first csRNA-seq libraries in cattle and bison demonstrate the applicability of our approach in livestock. Our analyses reveal that 5’ annotations of many Reference Sequence annotations (RefSeq) for cattle and bison, but not humans, are often inaccurate. This highlights the importance of the provided experimental data as, among others, accurate transcription start sites (TSSs) are critical for successful genome engineering approaches [16, 17].

## Methods

### Cell culture, siRNA & mRNA transfections

BT474 and A375 cells were grown at 37 °C with 5% CO_2_ in DMEM (Cellgro) supplemented with 10% FBS (Gibco), and 50 U Penicillin and 50 μg Streptomycin per ml (Gibco). For total RNA isolation, cells were washed once with ice cold DPBS (Gibco), rested on ice for 5 min, washed one more time with ice cold DPBS, and then lysed in 1 ml TRIzol Reagent (Thermo Fisher Scientific). RNA was isolated as described by the manufacturer.

Animal samples were obtained from healthy, live experimental cattle that belong to and were housed at Washington State University, Pullman, WA and bison at University of Wyoming, Laramie, WY. Animal care and use followed approved national and local regulations and standards. For sampling, animals were briefly restrained in a chute with the head contained in the head gate and 2.8 ml blood was collected by venipuncture (jugular) using PAXgene Blood RNA tubes (BD Bioscience). Samples were transported on ice to the laboratory for processing. RNA was isolated using the PAXgene Blood miRNA Kit (Qiagen) as described by the manufacturer.

### sRNA and csRNA-seq library generation

Small and capped small RNA [13] libraries were generated exactly as described [18]. Small RNAs of ∼ 15–60 nt were size selected from total RNA by denaturing gel electrophoresis. 10% of these RNAs were decapped and polyphosphates reduced to monophosphates using RppH (NEB) to sequence all small RNAs.

The remainder of the size selected sRNAs was enriched for 5’-capped RNAs. Monophosphorylated RNAs were selectively degraded by one hour incubation with Terminator 5´-Phosphate-Dependent Exonuclease (Lucigen). Subsequently, RNAs were 5’ dephosporylated through 90 min incubation in total with thermostable QuickCIP (NEB) in which the samples were briefly heated to 75 °C and quickly chilled on ice at the 60 min mark. Small RNA and csRNA-seq libraries were prepared using the NEBNext Small RNA Library Prep kit with an additional RppH step [19], amplified for 13 cycles, size selected again on a 10% TBE gel for ∼ 130–180 bp (118 bp adapter length), and sequenced SE80 on either the Illumina NextSeq 2000, PE100 on the Illumina NovaSeq 6000, or PE75 on the AVITI. Only Read 1 (R1) was used in analyses.

### AVITI library conversion for sequencing

Libraries prepared from human BT474 cells as well as cattle and bison blood were diluted (8.4–11.2 fmole per flow cell) and loaded directly to the instrument for circularization on the flow cell surface by the AVITI system using the new Cloudbreak Freestyle chemistry. This process does not incorporate additional sequence to the final library.

Libraries prepared from human A375 cells were converted for sequencing on the AVITI by following the currently standard Rapid Adept PCR-free protocol (Element Biosciences, #830-00007, provided also in the supplement of this paper as “Supplemental protocol”).

In brief, two A375 libraries were pooled and 0.15 pmole linear library was denatured and hybridized to a splint oligo mix. Circularization was achieved by ligation of both library ends to a 48 nt backbone oligo sequence to form a ssDNA circular molecule. Residual linear library and splint oligo are enzymatically digested, and the reaction is stopped with an EDTA solution. This protocol utilizes a stop solution over a bead-based cleanup to prevent loss of the carefully size selected sRNA and csRNA-seq libraries.

All linear libraries were quantified by Qubit dsDNA HS assay (Thermo Fisher Scientific) paired with fragment size analysis using Tapestation D5000 High Sensitivity screentapes (Agilent). Circular libraries were quantified using a qPCR assay as part of the Rapid Adept PCR-free protocol (Supplemental protocol, Element Biosciences, #830-00007).

### Sequencing

Illumina NextSeq 2000 sequencing was performed at the Washinton State University Molecular Biology and Genomics Core and NovaSeq S6000 sequencing at UC San Diego’s IGM core.

AVITI sequencing was performed at Element Biosciences (San Diego, CA). Libraries prepared from human BT474 cells, bison, and cattle samples were sequenced using Cloudbreak Freestyle chemistry kits (Element Biosciences, #860 − 00015) with the modified recipe for short fragments (Supplemental protocol, Element Biosciences, #830-00007). Libraries prepared from human A375 cells were sequenced using the Cloudbreak chemistry kit (Element Biosciences, #860-00004).

Custom sequencing primers were added for Read 2 (R2) and Index 1 on the AVITI system to all sequencing runs. Primers were ordered from IDT with HPLC purification (Read2: 5’- GTGACTGGAGTTCCTTGGCACCCGAGAATTCCA-3’, Index1: 5’-TGGAATTCTCGGGTGCCAAGGAACTCCAGTCAC-3’) and spiked-in to the existing sequencing primer tubes at a final concentration of 1 μM following the AVITI user guide (Element Biosciences, #MA-00008).

### Data analysis

Small RNA-seq and csRNA-seq sequencing reads were trimmed of their adapter sequences using HOMER2, and sequences shorter than 20 nt discarded to ensure reliable alignment to the human genome (homerTools trim − 3 AGATCGGAAGAGCACACGTCT -mis 2 -minMatchLength 4 -min 20 {raw read}) [14]. To achieve equal read depth, fastq files were subsampled using SeqKit’s sample (version 2.5.1) [20] before alignment to the appropriate reference genome: STAR for human (STAR --genomeDir {index} --runThreadN 20 --readFilesIn {input} --outFileNamePrefix {output}. --genomeLoad NoSharedMemory --outSAMattributes NH HI AS NM MD --outSAMstrandField intronMotif --outMultimapperOrder Random --outSAMmultNmax 1 --outFilterMultimapNmax 10000 --limitOutSAMoneReadBytes 10000000) [21] and Hisat2 for livestock (hisat2 -p 30 --rna-strandness RF --dta -x {index} -U {input} -S {output}.Aligned.out.sam 2> {output}.stats) [22], given differences in annotation (.gtf) quality.

Alignment files (.sam) were converted into tag directories using HOMER2 (batchMakeTagDirectory.pl {sam_infofile.txt} -cpu 8 -genome {species genome} -omitSN -checkGC -single -r). The tagLengthDistribution.txt file in the completed tag directories contains the distribution of read lengths. Features (peaks), representing strand-specific loci with significant transcription initiation (Transcription Start Regions, TSRs) for csRNA-seq or expressed small RNAs for sRNA-seq, were defined using HOMER2’s findcsRNATSR.pl and findPeaks, respectively. A minimum read count of 20 per 10 million was required for regions to be considered in the analysis (findcsRNATSR.pl {csRNA} -o {output_dir} -i {sRNA} -genome {genome} -gtf {gtf} -ntagThreshold 20 -cpu 30, findPeaks {sRNA} -o {output_dir} -i {csRNA} -gtf {gtf} -style tsr -ntagThreshold 20) [13]. Small RNA-seq data were integrated into the csRNA-seq analysis to eliminate loci with csRNA-seq signal arising from non-initiating, high abundance RNAs captured by the method. Replicate experiments were combined for each condition before identifying features. Additional information and analysis tutorials are available at http://homer.ucsd.edu/homer/ngs/csRNAseq/index.html.

### Sequencing quality control metrics

Statistics were summarized using Bases2Fastq and FastQC [23] to assess %GC, duplicate rate, average length, and median length, and Seqtk [20] to assess percent Q30, percent Q40, and mean quality score. In the last column, SAMtools [24] was used to calculate the mismatch rate (number of mismatches / bases mapped); However, this error rate calculation holds some bias because the reference genome used for alignment is not an exact match for the samples from the human cell lines.

### Differential expression

The previous TSRs, which were combined across the two replicates for each condition, were used to create new merged files for pairwise comparisons using HOMER’s mergePeaks tool (mergePeaks {condition 1} {condition 2} -strand > mergedTSRs.txt) [25]. Raw read counts were then quantified for each of the comparisons between conditions (annotatePeaks.pl {mergedTSRs} {genome} -gtf {gtf} -strand + -fragLength 1 -raw -d {tag directories} > raw_counts.txt). The resulting output was then analyzed using DESeq2 to calculate the rlog variance stabilized counts and identify differentially regulated TSRs [26]. For comparison, differentially expressed TSRs were also calculated using edgeR [27].

To visualize the overall difference in expression levels, the HOMER annotatePeaks.pl command used previously to quantify raw counts was reused to get the rlog-transformed counts by replacing the “-raw” argument with “-rlog” [14]. These normalized counts were used for the scatterplots.

### Read histograms

RefSeq TSRswere extracted from .gtf files using “parseGTF.pl {gtf} tss > RefSeqTSRs.txt”. Histograms showing experimental TSS to RefSeq TSS were created using “annotatePeaks.pl {RefSeqTSRs} {genome} -p {experimentalTSRs} -size 500 -hist 1 -strand + > distance_to_RefSeqTSS.txt” from HOMER.

### Motif analysis

The analyis of the core promoter elements (the TATA box and the Initiator) for our experimental TSRs and RefSeq TSRs was performed using HOMER’s annotatePeaks.pl tool (annotatePeaks.pl {TSRs} {genome} -size 150 -hist 1 -m {motif} > motif_hist.txt) [25]. This tool was also used to find the nucleotide frequency plots (annotatePeaks.pl {TSRs} {genome} -size 1000 -hist 1 -di > nt_hist.txt).

### Modified bison GTF

The first column of the bison gtf file underwent an ID update to achieve consistency with the fasta genome. A key was generated, linking the old IDs to their new counterparts in the genome (Table S1). Chromosome IDs (1–29, Y) were directly replaced with their corresponding accession numbers found in the original study’s NCBI BioProject repository (PRJNA677946) [28]. For IDs labeled “scaffold_XXX,” a specific transformation was applied: 10,000,000.1 was added to the number following the underscore, and the resulting number was prefixed with “JAEQBK0.” The modified bison .gtf (GSE267848_modified_Bison_bison_liftoff.ARS-UCSC_bison1.0.gtf.gz; GEO supplementary file) was then created using the following custom code.

name_key_df = pd.read_csv(“Table_S1.csv”, dtype={“old_id”: “string”})

name_key = dict(zip(name_key_df[“old_id”], name_key_df[“new_id”]))

cols = [“gtf_id”, “source”, “feature”, “start”, ‘“end”’, “score”’, “strand”’, “frame”, “attributes”].

gtf = pd.read_csv(“Bison_bison_liftoff.ARS-UCSC_bison1.0.gtf”, sep = “\t”, header = None, names = cols, dtype={“gtf_id”: “string”})

modified_gtf = gtf.assign(gtf_id = gtf[“gtf_id”].map(name_key))

modified_gtf.to_csv(“modified_Bison_bison_liftoff.ARS-UCSC_bison1.0.gtf”, sep = “\t”, index = False, header = False, quoting = 3).

## Results

### Uniform small RNA coverage among Illumina and AVITI sequencing technologies

**Fig. 1:**
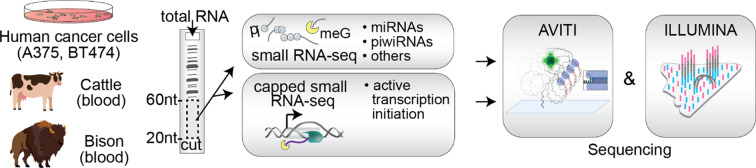
**Study design.** Small RNAs were purified from total RNA isolated from human cancer cell lines (BT474, A375), cattle and bison blood. Libraries containing all small RNAs (sRNA-seq) as well as 5’meG cap-enriched small RNAs that are associated with actively initiating RNA polymerase II (csRNA-seq) of size 20–60 nt were sequenced on the AVITI and Illumina NGS platforms

To overcome prior limitations, we developed and tested an optimized and accelerated protocol for AVITI short read circularization-based sequencing compatible with both the current Rapid Adept and the next generation Cloudbreak Freestyle AVITI chemistries (Fig. S1) using human cancer cells and blood from cattle and bison, two animals of agricultural importance, and compared the results to Illumina. We generated small RNA (20–60 nt) libraries, containing all mono, di, tri, 5’ capped or otherwise 5’ modified sRNAs [29], and csRNA-seq libraries, which captured capped short RNAs that are associated with active initiation of RNA polymerase II promoters and enhancers [12–14, 30–32] from each sample. Libraries were then sequenced on the Illumina NovaSeq 6000, Illumina NextSeq 2000, or the Element Biosciences AVITI platforms (Fig. 1), downsampled to an equal number of reads (Table S2), and subsequently compared.

To assess potential biases, we first compared the read length distribution of aligned fragments in human BT474 and A375 cancer cells in replicate (please see methods). Samples were circularized either directly on the AVITI flow cell or on benchtop prior to sequencing, for the BT474 and A375 cells. Any technical differences between the Illumina and AVITI methods, such as bridge amplification versus rolling circle amplification, are expected to result in a linear size bias relationship across all samples. By contrast, differences arising from experimental replicates could be non-linear.

**Fig. 2:**
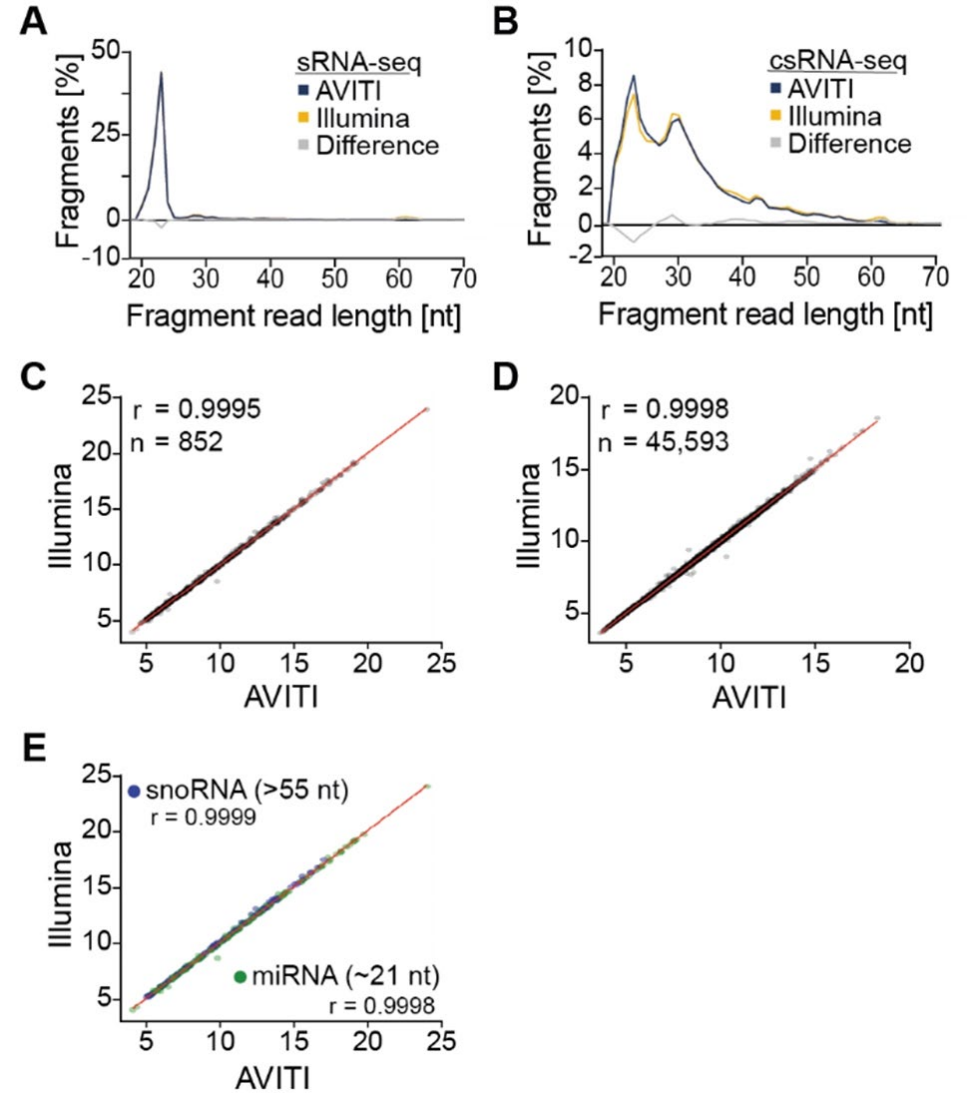
**Uniform sequencing of small and capped small RNA-seq libraries on the Illumina and AVITI platforms.** **A**. Read length distribution plots of BT474 small RNAs sequenced natively on the Illumina and the AVITI platform using the Cloudbreak Freestyle method. The area under each line sums to a total of 100%. Differences between Illumina and AVITI are plotted in grey. **B**. Read length distribution plots of BT474 cells capped small RNAs. **C**. Scatterplot comparing the expression level (rlog transformed read counts) of small RNAs and **D**. capped small RNAs using the Illumina and AVITI platform. **E**. Comparison of the detection of small RNA types of different lengths (miRNAs: 21–24; snoRNAs: 55–61 F)

Observed differences in size distribution among the two sequencing methods did not correlate with aligned fragment length for sRNA or csRNA-seq (paired t-test, *p* > 0.99, Fig. 2A, B, Fig. S2 A, B). Concordantly, expression levels of > 852 sRNAs as well as transcripts from > 45,593 active regulatory elements, such as promoters and enhancers, captured by csRNA-seq, were highly similar across sequencing platforms (*r* = 0.9995 and *r* = 0.9998, respectively, Fig. 2C, D, Fig. S3 A, B; please see methods for quantification of expression). Indeed, differences were more pronounced between replicates on each platform than between Illumina and AVITI sequencers (Fig. S3 C, D).

Analyzing differentially expressed loci between platforms using DEseq2 [26] revealed only 4 downregulated in Illumina data, and 19 in AVITI data for the BT474 cell line (log_2_FC, FDR 0.05; 7 and 26 downregulated and upregulated loci were identified by edgeR). Equivalently high correlations were observed among nucleotide frequencies (Fig. S2 C, D), miRNAs (21-24 nt in length) and the > 55 nt long small-nucleolar (sno)RNAs [33] (Fig. 2E, Fig. S3 E). Furthermore, the top 10 sRNAs identified from the two platforms resulted in similar expression levels that were not statistically different (Table S3).

Similarly, no significant differences were observed between sequencing platforms in the average GC content of samples (paired t-test, *p* = 0.414), while the average trimmed read length of AVITI reads (25 bp) was slightly lower than Illumina (28 bp) (two sample t-test, *p* < 0.01, Table S2). Average estimated quality across all bases of trimmed reads was higher for AVITI reads than Illumina (Q = 42.4 vs. Q = 35.4, two sample t-test, *p* < 0.01, Table S2). Alignment mismatch rates were equivalent (0.09% AVITI, 0.10% Illumina, two sample t-test, *p* = 0.460). Together, these data argue that short fragments are efficiently captured by both sequencing platforms.

### Accurate coverage of small RNAs and active TSSs in cattle and bison reveals a need to improve annotations in livestock

To test our protocol and compare the sequencing technologies in species of agricultural importance we next performed sRNA and csRNA-seq on blood collected from cattle and bison. Consistently, we observed similar size profiles and sequencing distributions with either Illumina or AVITI sequencing methods (paired t-test, *p* = 0.99, Fig. S4).

**Fig. 3:**
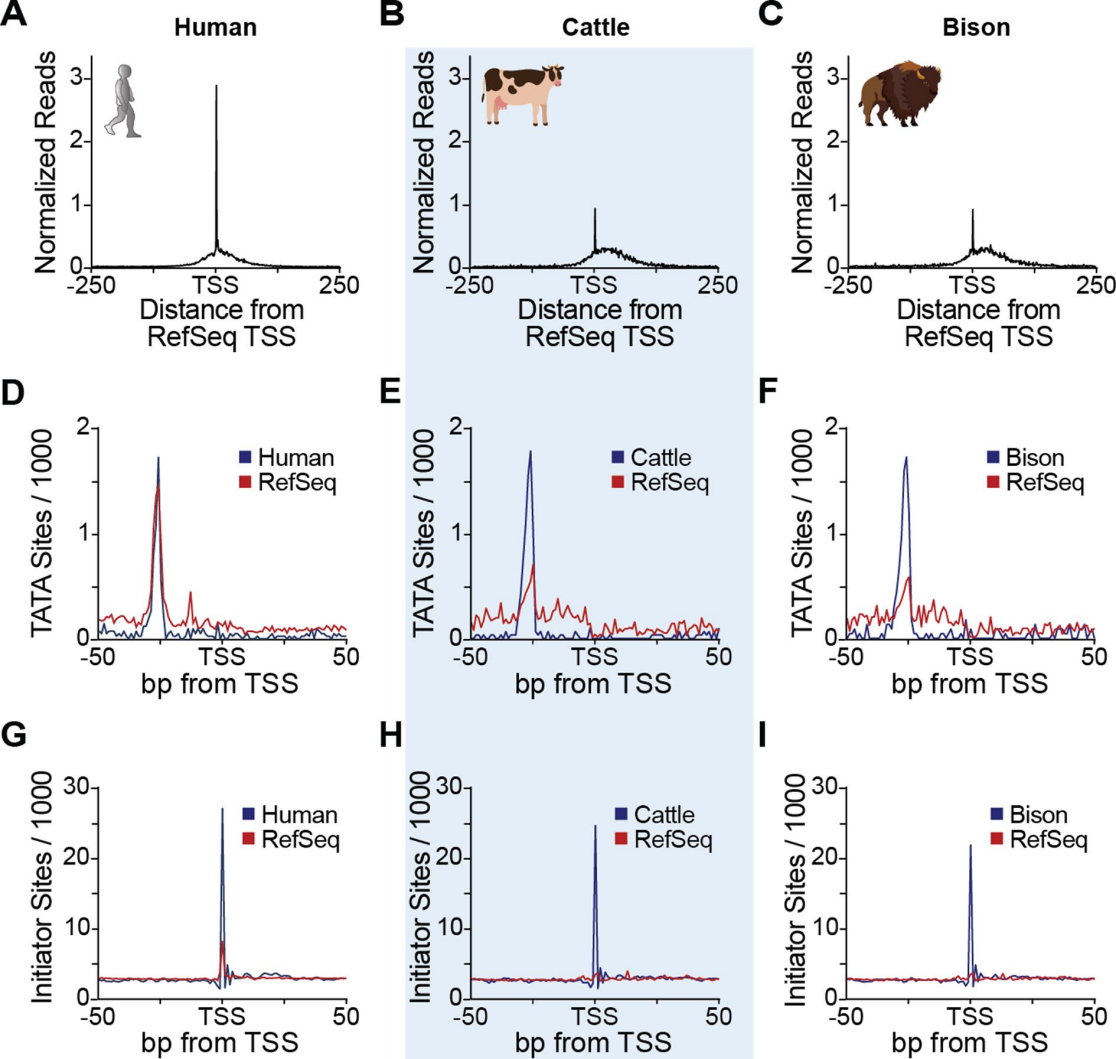
**csRNA-seq facilitates improved genome annotations.** (**A**) Comparison of experimentally defined TSSs from human BT474 cancer cells, (**B**) cattle, and (**C**) bison by csRNA-seq sequenced using AVITI relative to the RefSeq annotation. (**D**) Comparison of the frequency of TATA box sites per 1000 bp between our experimental TSS and RefSeq for human, (**E**) cattle, and (**F**) bison. (**G**) Comparison of the frequency of Initiator sites per 1000 bp between our experimental TSS and RefSeq for human, (**H**) cattle, and (**I**) bison

During our analyses, we observed notable inconsistencies among our experimental TSS data and many annotated 5’ ends of genes in cattle and bison but importantly, not human (Fig. 3A-C, Fig. S5 A, B; human: GCF_000001405.40, cattle: GCF_002263795.2, bison: modified_GCA_018282365.1). Many TSSs were within 100 bp of RefSeq 5’ annotations (39.7% in cattle and and 45.5% in bison), suggesting quality annotation of coding regions. However, genic 5’ ends and promoters were often inaccurate. Since accurate 5’ annotations are an essential part of many analyses, including genome engineering [16, 17] or decoding gene regulatory programs [14, 34], and to our knowledge, our study presents the first nascent TSS data for cattle and bison, we investigated the differences further.

It is important to note that TSSs often change across tissues and cell types, making it difficult for a single annotation to accurately capture all this variation [16]. However, computational prediction of gene 5’ ends, and to some extent even the mapping of mature processed transcripts rather than nascent ones, has been shown to introduce annotation inaccuracies [35, 36]. To address these discrepancies, we analyzed unbiased biological features, such as core promoter elements and TSS-proximal nucleotide frequencies. This analysis demonstrated a clear improvement in the accuracy of our experimentally determined TSSs compared to RefSeq annotations.

Core promoter elements anchor and dictate the site of RNA polymerase initiation. Consequently, they are highly positionally enriched: the TATA box at -31 to -26 and the Initiator from − 2 to + 4, relative to the TSS [37, 38]. In addition to the increased information content in the TSS-proximate nucleotide frequencies (Fig. S6), the TATA box and Initiator core promoter elements were found at the expected positions in the human RefSeq and in our experimental TSS data, but not in the cattle and bison RefSeq annotations (Fig. 3D-I, Fig. S5 C, D). Together, these observations provide an independent validation for our experimental TSS data, stress their importance and the need to improve the genome annotations in these agriculturally important species.

## Discussion

Here, we provide a protocol for sequencing short sequences such as miRNAs, snoRNAs, or csRNAs on the AVITI platform and demonstrate uniform results to Illumina sequencing. Given their similar properties, it is likely that other classes of small RNAs like siRNAs, piRNAs, repeat-associated small interfering RNAs (rasiRNAs) [5], tRNA halves (tiRNA), tRNA-Derived Fragments (tRF), as well as many others [6–8], can also be readily assayed.

Sequencing on the AVITI requires library circularization, which can be performed on the benchtop prior to sequencing, or directly on the flow cell surface post-library loading (Fig. S1). Both benchtop circularization (A375 samples) and flow cell surface circularization (BT474, bison, and cattle samples) methods were tested and shown to be concordant with Illumina data (Fig. S2, Fig. S4). Therefore, both methods can be used. While benchtop circularization allows for additional library quality control, flow cell circularization simplifies the workflow and reduces hands on time and sample input requirements (Fig. S1). We hence recommend flow cell circularization for routine applications. These findings underscore the adaptability of the AVITI rolling circle amplification strategy to a wide range of library sizes, from inserts > 1000 bp (Carroll et al., *in preparation*) to ≤ 20 bp fragments, as demonstrated here.

In addition, our study generated sRNA and nascent TSS data for human BT474 and A375 cells, as well as the first data of such kind for cattle and bison, thereby enriching publicly available resources for the scientific community. Analysis of these data not only demonstrated the utility of csRNA-seq and AVITI sequencing in livestock but also their necessity. While annotated 5’ ends of genes largely agreed with nascent TSSs in well studied organisms, like humans, clear differences were observed in cattle and bison. Our experimental TSSs improve upon RefSeq by revealing biological features such as position-constrained core promoter elements [39, 40]. This is also important as accurate TSSs and targeting of the promoter is critical for genome engineering efforts [16, 41].

The use of multivalent avidites to detect bases on AVITI further resulted in higher sequencing quality metrics compared to Illumina (Table S2). However, sequence polymorphisms among the utilized cell lines or individual bison and cattle as well as the specific reference genomes, dominated alignment rates, making this difference negligible for sRNAs. Therefore, our study not only provides a new protocol to sequence small sequences polymers on the AVITI and nascent TSSs for bison and cattle, but also highlights the possibility of studying these small molecules on either the AVITI or Illumina platform, increasing flexibility for researchers and, by demonstrating uniformity, validating both methods.

## Conclusions

Our demonstration of an accelerated and optimized circularization and sequencing protocol bridges the advantages of AVITI sequencing and methods that rely on sequencing short fragments such as sRNA-seq, miRNA-seq, or csRNA-seq.

## Electronic supplementary material

Below is the link to the electronic supplementary material.

Supplementary Material 1

Supplementary Material 2

## Data Availability

All next-generation-sequencing (NGS) data are available at NCBI Gene Expression Omnibus with the accession number GSE267848. Other data that support the findings of this study are available from the corresponding author upon request.

## Abbreviations

cfDNA: Cell-free DNA
csRNA: Capped small RNA
miRNA: MicroRNA
ncRNA: Non-coding RNA
NGS: Next-Generation Sequencing
piRNA: Piwi-interacting RNA
rasiRNA: Repeat-associated small interfering RNA
siRNA: Small interfering RNA
snoRNA: Small nucleolar RNA
sRNA: Small RNA
tiRNA: TRNA halves
tRF: TRNA-Derived Fragments
tRNA: Transfer RNA
TSR: Transcription start region
TSS: Transcription start site
