# Supplementary material for: Efficient small fragment sequencing of human, cattle, and bison miRNA, small RNA, or csRNA-seq libraries using AVITI
Source: BMC Genomics. 2024 Nov 29;25:1157. doi: 10.1186/s12864-024-11013-7 (PMC11606011; doi:10.1186/s12864-024-11013-7)
Supplement: Supplementary file 1 — Supplementary Material 1 [file 12864_2024_11013_MOESM1_ESM.docx]

**SUPPLEMENTAL FIGURES**

**Efficient small fragment sequencing of human, cattle, and bison miRNA, small RNA or csRNA-seq libraries using AVITI**

Anna L. McDonald ^a^, Andrew M. Boddicker ^b^, Marina I. Savenkova ^a^, Ian M. Brabb ^a^, Xiaodong Qi ^b^, Daniela D. Moré ^c,d^, Cristina W. Cunha ^c,d^, Junhua Zhao ^b^, Sascha H. Duttke ^a*^

^a^ School of Molecular Biosciences, College of Veterinary Medicine, Washington State University, Pullman, WA, USA

^b^ Element Biosciences, San Diego, CA, USA

^c^ Animal Disease Research Unit, Agricultural Research Service, United States Department of Agriculture, Pullman, WA 99164, USA.

^d^ Department of Veterinary Microbiology and Pathology, Washington State University, Pullman, WA 99164, USA


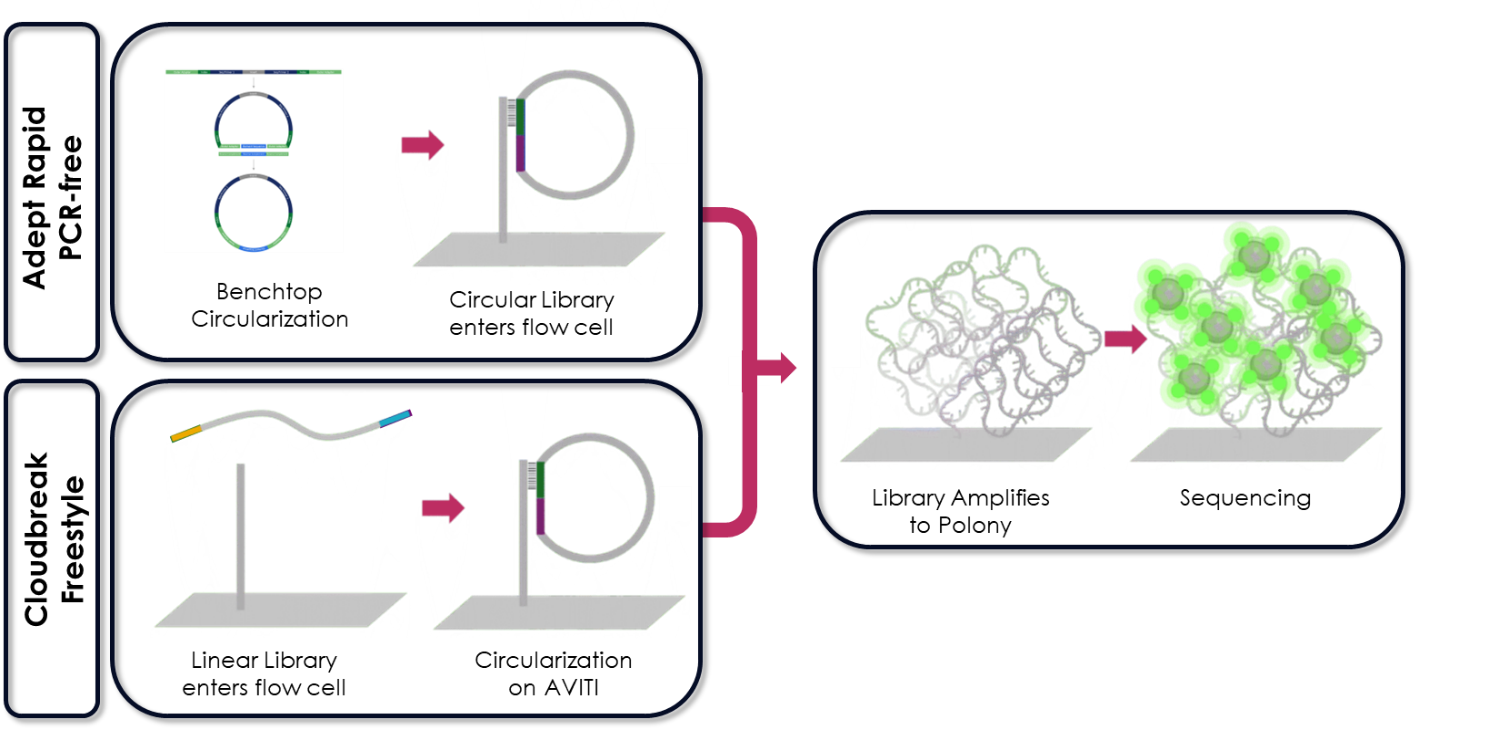


**Fig. S1:** **Overview of optimized small DNA fragment sequencing workflow using AVITI.** Both circularization methods for AVITI sequencing require the same standard sequencing library preparation as input. Adept Rapid PCR-free uses benchtop circularization, requires 10 minutes of hands-on time and 40 minutes of total assay time, including the recommended additional quality control prior to sequencing. Cloudbreak Freestyle circularizes libraries on AVITI and does not require any additional library modification or quality control before sequencing.


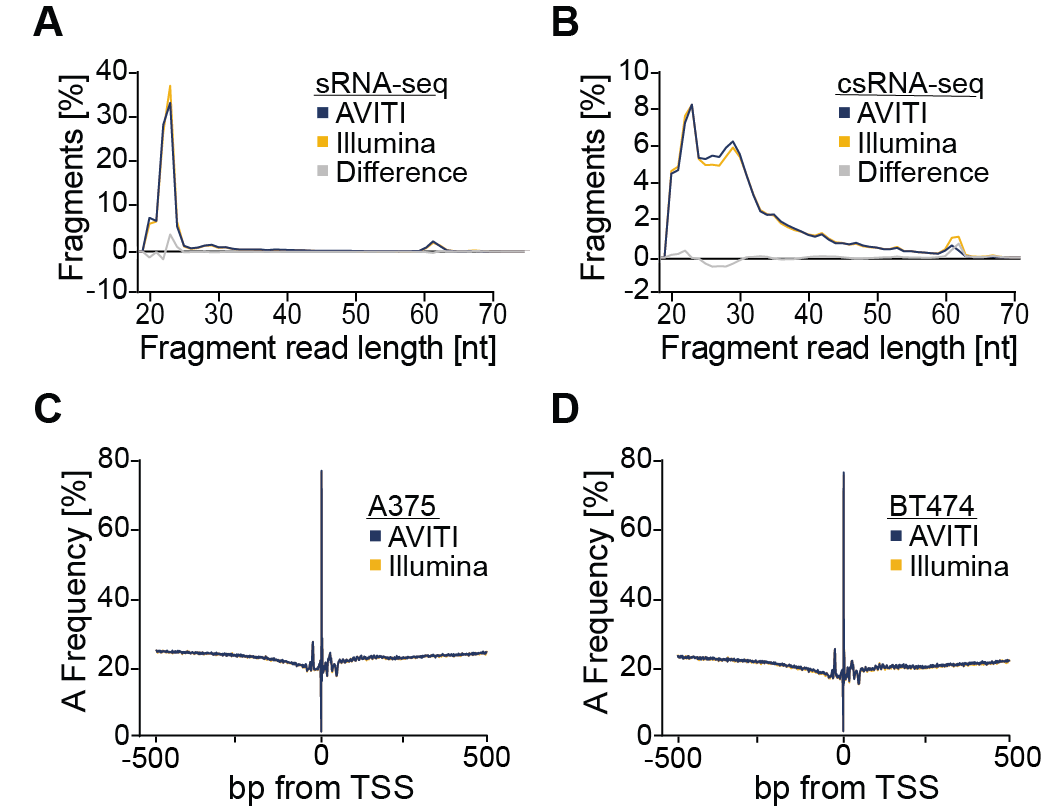


**Fig. S2: Additional comparisons of fragment length and nucleotide biases.** **A**. Read length distribution plots of A375 small RNAs sequenced on the Illumina and after benchtop circularization (Adept Rapid PCR-free) on the AVITI platform. The area under each line sums to a total of 100%. Differences between Illumina and AVITI are plotted in grey. **B**. Read length distribution plots of A375 capped small RNAs. **C**. An adenine nucleotide frequency plot of TSSs from Illumina and AVITI for A375 and **D.** BT474 human cancer cells.


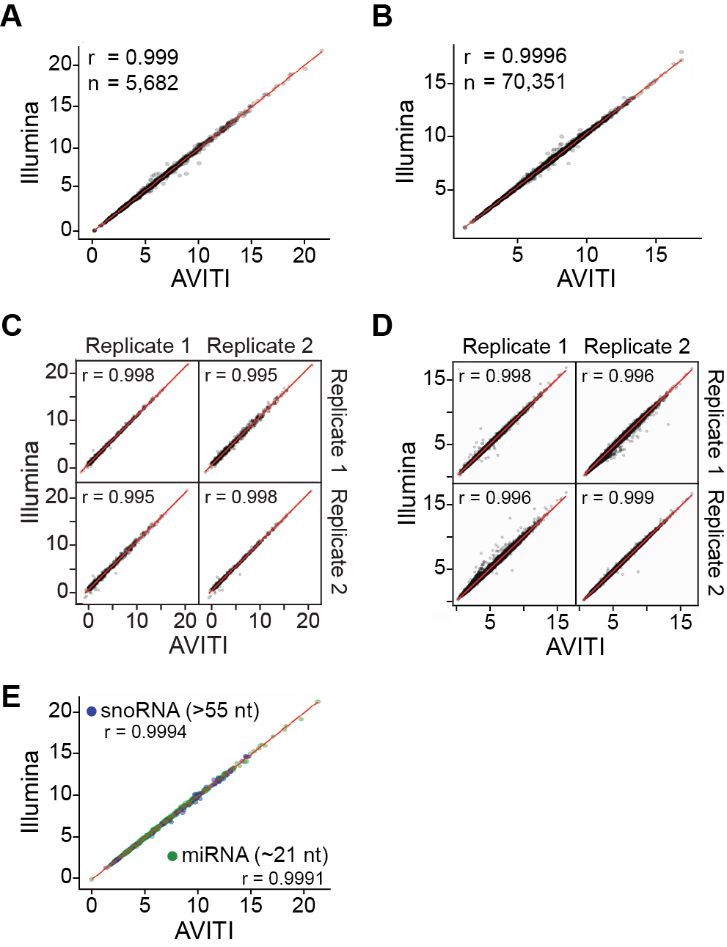


**Fig. S3: Correlation among libraries sequenced using AVITI and Illumina. A.** Scatterplot comparing the expression level of A375 small RNAs and **B**. capped small RNAs using the Illumina and AVITI platform. **C.** Scatterplot comparing the expression level among replicates of BT474 small RNAs and **D.** capped small RNAs using the Illumina and AVITI platform. **E**. Comparison of the detection of A375 small RNA types of different lengths (miRNAs: 21-24; snoRNAs: 55-61).


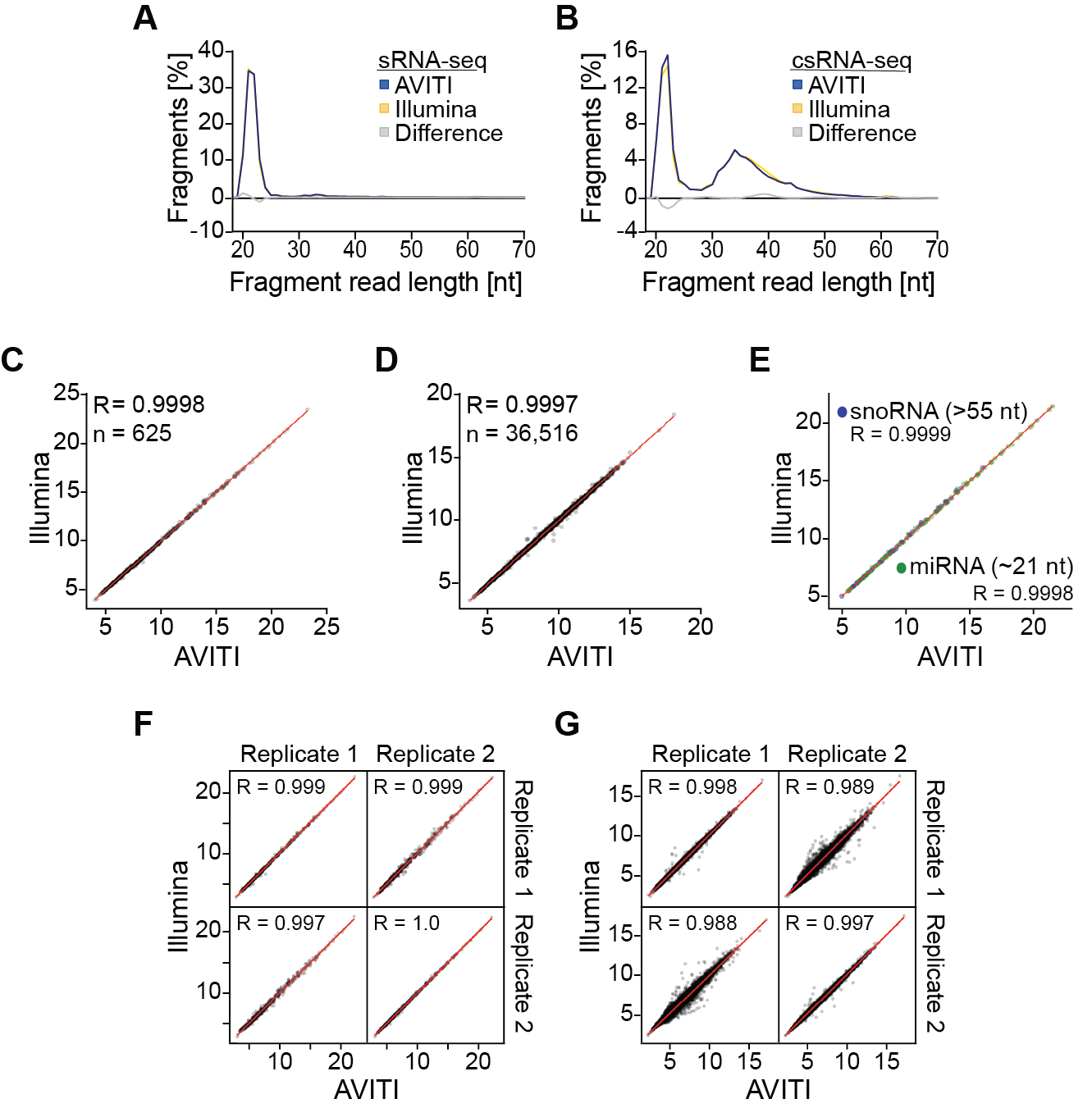


**Fig. S4: Correlation among libraries generated for cattle sequenced using AVITI and Illumina. A.** Read length distribution plots of cattle small RNAs sequenced on the Illumina and AVITI platform. Each graph sums up to 100% except the grey graph which denotes the difference between Illumina and AVITI. **B.** Read length distribution plots of cattle capped small RNAs. **C**. Scatterplot comparing the expression level of small RNAs and **D**. capped small RNAs using the Illumina and AVITI platform. **E**. Comparison of the detection of small RNA types of different lengths (miRNAs: 21-24; snoRNAs: 55-61). **F.** Scatterplot comparing the expression level among replicates of cattle small RNAs and **G.** capped small RNAs using the Illumina and AVITI platform.


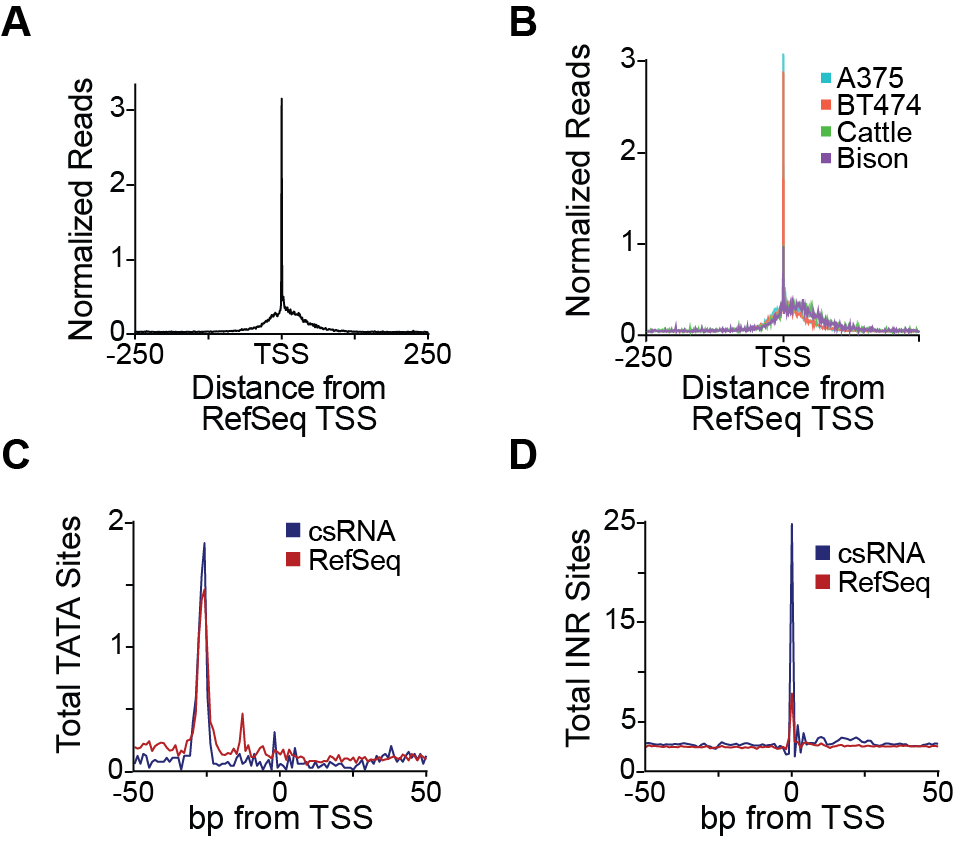


**Fig. S5: csRNA-seq could be used to improve 5’ annotations of livestock genes. A.** Comparison of experimentally defined TSSs from A375 by csRNA-seq relative to the human RefSeq annotation and **B.** all comparison of experimentally defined TSS from the respective RefSeq. **C.** Comparison of the frequency of TATA box sites and **D.** Initiator sites per 1000 bp between our experimental TSS and RefSeq for A375**.**


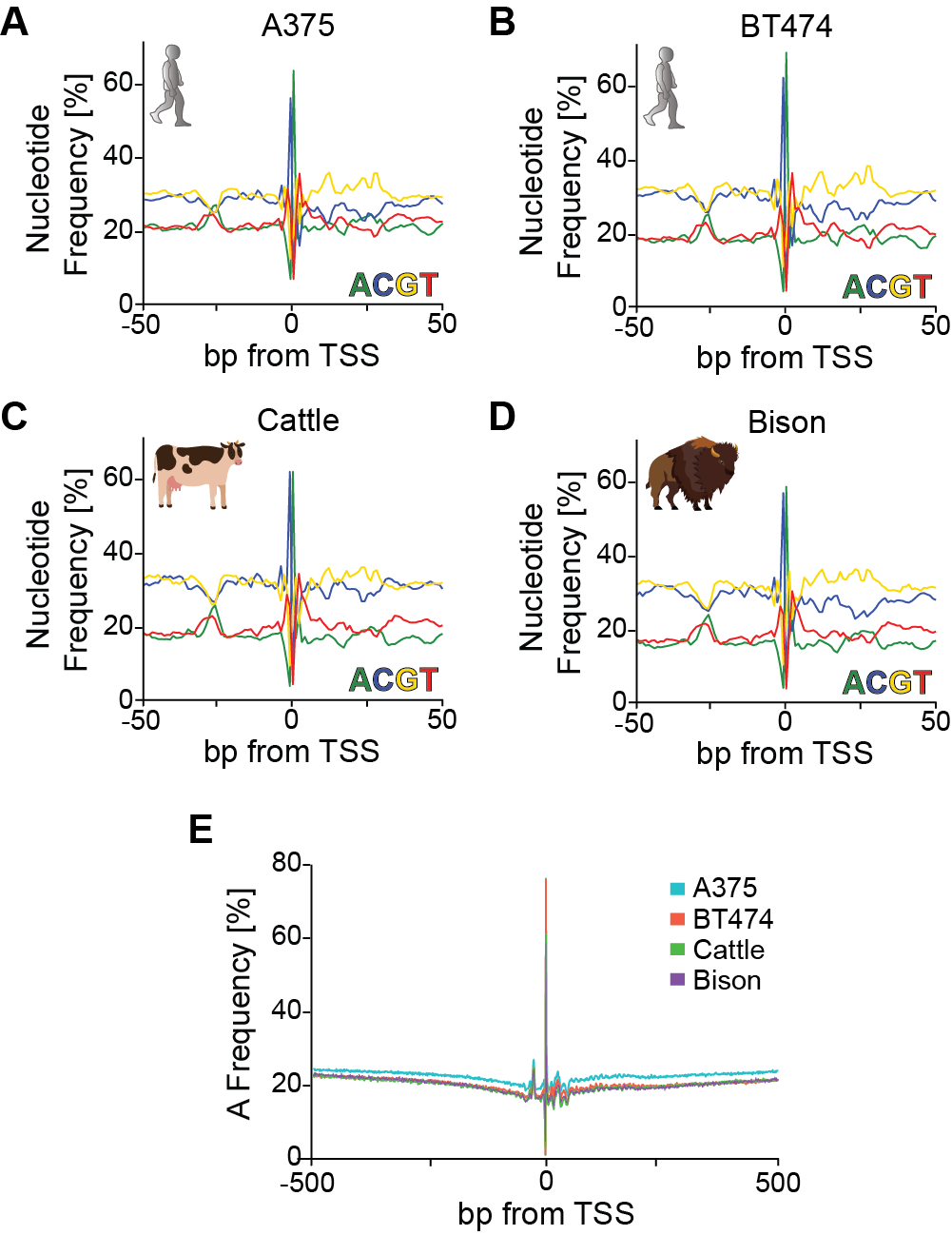


**Fig. S6: Nucleotide frequencies near human, cattle and bison transcription start sites experimentally defined by csRNA-seq. A.** Nucleotide frequency plots of TSSs in human A375 cancer cells, **B.** human BT474 cancer cells, **C.** cattle, and **D.** bison. **E.** Combined adenine nucleotide frequency plot for each species and cell line.
